# LESSONS LEARNED FROM DIGITAL EQUALITY RESEARCH WITH COMMUNITY PARTNERS

**DOI:** 10.1093/geroni/igad104.1720

**Published:** 2023-12-21

**Authors:** Eleanor Batista-Malat, Sindy Lomeli, Alexandria Capolino, Skye Leedahl, Kathleen Wilber

**Affiliations:** University of Southern California, Los Angeles, California, United States; University of Southern California, Los Angeles, California, United States; University of Rhode Island, Kingston, Rhode Island, United States; University of Rhode Island, Kingston, Rhode Island, United States; University of Southern California, Los Angeles, California, United States

## Abstract

Despite strong interest among policymakers and service providers and a pipeline of Federal funding for programs that address digital inequalities in older adults, there is limited research on the challenges and outcomes of programs designed to reduce the digital divide. To better understand these issues, we compared and contrasted challenges and lessons learned administering digital inclusion projects in California and Rhode Island. In California, challenges included the complexity of state and local interface, managing public-private partnerships, and deciding on and communicating roles and responsibilities through the required formal channels. In Rhode Island, timing and communication were challenging because universities and community partners operate on different calendar systems and cater to different demands. For both programs, ensuring that training and technical support were available, accessible, and appropriate for diverse populations of older adults was a challenge. However, the training and support approaches were different as California leveraged private and non-profit partnerships while Rhode Island used university student mentors. Further, the rollout and data collection needed to evaluate the programs created barriers and slowed the process, including partners’ language capabilities not matching the participants’ and the time-consuming nature of data collection via telephone. Nevertheless, in both states, research opportunities afforded by collaborating with state and local agencies and private sector providers were invaluable. Partnerships allowed older adults to participate in research via a trusted organization with linguistic and cultural sensitivity, and researchers accessed underserved populations less likely to participate in research.

